# Cognitive Interference Alignment Schemes for IoT Oriented Heterogeneous Two-Tier Networks

**DOI:** 10.3390/s18082548

**Published:** 2018-08-03

**Authors:** Run Tian, Lin Ma, Zhe Wang, Xuezhi Tan

**Affiliations:** 1 School of Electronics and Information Engineering, Harbin Institute of Technology, Harbin 150001, China; tianrun@msu.edu (R.T.); malin@hit.edu.cn (L.M.); 2 School of Engineering, Michigan State University, East Lansing, MI 48824, USA; wangzh34@msu.edu

**Keywords:** internet of things, interference alignment, heterogeneous networks, cognitive radio

## Abstract

This paper considers interference management and capacity improvement for Internet of Things (IoT) oriented two-tier networks by exploiting cognition between network tiers with interference alignment (IA). More specifically, we target our efforts on the next generation two-tier networks, where a tier of femtocell serving multiple IoT devices shares the licensed spectrum with a tier of pre-existing macrocell via a cognitive radio. Aiming to manage the cross-tier interference caused by cognitive spectrum sharing as well as ensure an optimal capacity of the femtocell, two novel self-organizing cognitive IA schemes are proposed. First, we propose an interference nulling based cognitive IA scheme. In such a scheme, both co-tier and cross-tier interferences are aligned into the orthogonal subspace at each IoT receiver, which means all the interference can be perfectly eliminated without causing any performance degradation on the macrocell. However, it is known that the interference nulling based IA algorithm achieves its optimum only in high signal to noise ratio (SNR) scenarios, where the noise power is negligible. Consequently, when the imposed interference-free constraint on the femtocell can be relaxed, we also present a partial cognitive IA scheme that further enhances the network performance under a low and intermediate SNR. Additionally, the feasibility conditions and capacity analyses of the proposed schemes are provided. Both theoretical and numerical results demonstrate that the proposed cognitive IA schemes outperform the traditional orthogonal precoding methods in terms of network capacity, while preserving for macrocell users the desired quality of service.

## 1. Introduction

Next generation wireless networks are expected to have an “everything, everywhere and always connected” future, where the end users shift from individuals to things. The concept of Internet of Things (IoT) has been proposed to cater for a massive number of smart devices and provide high-speed data services. To cope with the ever-increasing wireless traffic demands induced by IoT, a hierarchical approach to network deployment with a densely populated femtocell base station has been proposed. Applications, such as cloudified mobile networks [1], virtual infrastructure [2], and industrial communication, also exploit heterogeneous architecture to support ubiquitous, flexible, and reliable connectivity [3]. Such proliferation of wireless base stations and data offloading strategies are to be expected in the near future, presumably yielding the two-tier approach to network design [4]. More specifically, a tier of IoT oriented femtocell base stations coexists with a tier of pre-existing macrocell base stations (MBSs) and shares the licensed spectrum. This two-tiered deployment aims at breaking away from the traditional cellular layout to provide very high data rates for short-range IoT devices, and reduces the load on the macrocell network [5].

In the IoT network, smart devices are a low-power plug and play ones with rather small transmission ranges. Deployed by end users, the IoT femtocell usually lacks a predefined network infrastructure. Such architecture can reduce the load and congestion on the macrocell network, and enables the resources to be allocated to the truly mobile users [6]. On the other hand, with a huge volume of IoT devices connected to the existing macrocell based network, the IoT paradigm places an overwhelming demand on the spectrum resources. It is preferred for femtocell to share the licensed frequency band with the pre-existing macrocell network. However, cell splitting and spectrum sharing will most probably cause harsh cross-tier interference, which restricts the reusability of frequency resources and becomes a key limiting factor in developing the IoT technology [7]. To ensure the peaceful coexistence of macrocell and femtocell, as well as improving the performance of cell-edge users, effective interference management scheme design has recently attracted growing interest [8].

It is indicated that the things-oriented, Internet-oriented networks are meaningless if IoT objects are not equipped with cognitive capability [4]. To mitigate the cross-tier interference, a cognitive radio (CR) has been introduced into IoT oriented heterogeneous networks. The notion of cognitive empowered IoT femtocell was proposed to enable IoT devices as femtocell users to be aware of their environment, and can quickly adapt to the network variations by changing their transmission patterns [9,10]. In other words, the cognitive femtocell could sense the idle licensed spectrum, allowing the IoT devices to communicate with the base station over the underutilized frequency band. However, in traditional cognitive radio networks, cognitive users can utilize the licensed band only when the primary user is non-active. The capacity and accessibility will largely depend on the primary user activity [11]. Inspired by the interference management, the CR-based network can operate in an underlay paradigm. By limiting the performance degradation caused to the primary user under a predefined threshold, both primary and cognitive users can transmit their data simultaneously within the same frequency band [12]. Cognitive interference alignment (IA) is an emerging interference management technology that has received extensive research. The basic idea here is to fully exploit the precoding matrix of the cognitive transmitters, then align the cross-tier interference signals into a lower dimensional subspace. More specifically, in each primary receiver, the cognitive interference caused by IoT devices should be aligned into the subspace orthogonal to the primary link so that the desired signal can be recovered free of cross-tier interference [13].

A number of state-of-the-art cognitive IA algorithms have been proposed in the literature, so as to ensure the coexistence of such two tiers and enhance the effective utilization of the licensed spectrum. For example, in [14], Guler et al. proposed a selective interference alignment with user selection. By judiciously choosing the set of cognitive transmitters to be aligned at each primary receiver, the selective interference alignment achieved a trade-off between algorithm complexity and achievable capacity. In [15], Rezaei et al. considered the scenario with just one cognitive user and proposed a cooperative IA scheme. The proposed scheme minimizes the distance between the subspaces of the co-tier and cross-tier interferences, and aligns them into the same orthogonal subspace at each primary user. In [16], Guler et al. focus on the uplink interference management in heterogeneous cellular systems, and designed an IA based precoder. The precoding design was accomplished using successive semi-definite programming (SDP), aiming to minimize the cross-tier interference leakage while providing a suboptimal received signal to interference plus noise ratio (SINR). Although the successive SDP approximation reduces the complexity, the proposed algorithm requires cooperation from macrocell users (MUs) with the closest femtocell base station (FBS), and only achieves a fraction of the optimal system capacity. In [17], Maso et al. proposed a distributed cognitive interference alignment (DCIA) algorithm in the absence of cooperation between two tiers. The DCIA algorithm only considers the interference to the primary tier, which reduces the overhead of the channel estimation in tiered networks. In [18], for the case of a dense heterogeneous cellular layout, Debbah et al. proposed an orthogonal transmission precoding scheme named MU-VFDM. By exploiting the cooperation and designing the precoder as a cascaded linear structure, the MU-VFDM algorithm achieves a comparable performance to the homogeneous scenario with multi-user coding techniques.

As can be seen, existing cognitive IA schemes mainly focus on the cross-tier interference caused to primary users. There is no suppression on the interference from primary users to cognitive users, which may cause severe performance degradation. It is also significant to note that most existing schemes require several degrees of co-tier and cross-tier coordination, which involves dedicated bidirectional connection between the macrocell and femtocell. However, due to the unplanned deployment and self-organizing features of IoT networks, it may be impractical to provide the bidirectional cooperative links that meet the latency and delay requirements [19]. Moreover, for cooperative IA, rather than designing the precoder to achieve the optimal capacity, primary users must compromise its performance to help cognitive users to mitigate the cross-tier interference. This turns out to be a major limitation that cooperative IA may degrade the channel capacity of the primary links. It is also overlooking the principle that cognitive radio networks should be transparent to primary users without causing any undesired performance degradation to them [20].

Motivated by these observations, in this paper, we consider a two-tiered network comprised of a pre-existing macrocell tier and a cognitive IoT femtocell tier, and propose two novel self-organized cognitive IA schemes to manage the interference and improve the network capacity. The main contributions of this paper can be summarized as follows:

- To fully eliminate the interference, we first propose an interference nulling based cognitive IA (IN-CIA) scheme. In such a scheme, both co-tier and cross-tier interferences are perfectly aligned into the orthogonal subspace at each receiver, without causing any performance degradation on the network users;
- as we know, the interference nulling based IA algorithm can achieve its optimum only with negligible noise. By slightly loosening the interference-free constraint on the cognitive femtocell, we also present a partial cognitive IA (P-CIA) scheme that can further improve the network performance under low and intermediate SNR conditions;
- additionally, the feasibility condition, the proof of convergence, and the capacity analyses are derived to demonstrate the effectiveness of the proposed cognitive IA schemes; and
- note that the proposed cognitive IA schemes can be performed in an autonomous way that requires no explicit cooperation between the two tiers. Such schemes can satisfy the low-cost and self-organized features of IoT, and achieves a considerably high network capacity while causing no interference to the macrocell users.

Theoretical and numerical analyses are provided to show that the proposed cognitive IA schemes can realize the coexistence between the two tiers, yielding a significant sum-rate and spectral efficiency enhancements for a large range of signal to noise ratio (SNR) values.

The rest of the paper is organized as follows. In Section 2, the scenario and system model are introduced. In Section 3, two novel cognitive IA schemes are proposed. The feasibility condition, convergence, and capacity analyses are carried out in Section 4. Numerical simulation results are presented in Section 5, followed by the conclusions and future work given in Section 6.

For the notation throughout this paper, we let a lower case italic symbol (e.g., x) denote a scalar value, a lower case bold italic symbol (e.g., x) denote a vector, and an upper case bold symbol (e.g., X) denote a matrix. $I_{N}$ represents the N×N identity matrix, and $0_{M\times N}$ denotes an M×N zero matrix. The eigenvector of A corresponding to the *i*-th eigenvalue in ascending order is presented as $v_{A,i}$. tr{A}, rank{A}, and $A^{H}$ are the trace, rank, and conjugate transpose of matrix A, respectively. Moreover, ${\mathbb{C}}^{M\times N}$ denotes the space spanned by complex M×N matrices, and CN(a,A) stands for the complex Gaussian distribution with a mean, a, and a covariance matrix, A. E[·] denotes the statistical expectation. All vectors are defined to be columns, and in vector and matrix definitions, the subscript “*p*” refers to the primary tier.

## 2. System Model 

In this section, we first set up the system model of the IoT oriented two-tier cognitive network. Then, we show that the cross-tier interference induced by cognitive spectrum sharing will invalidate the full-IA algorithm, which motivates us to design cognitive empowered IA schemes.

**Figure 1:**
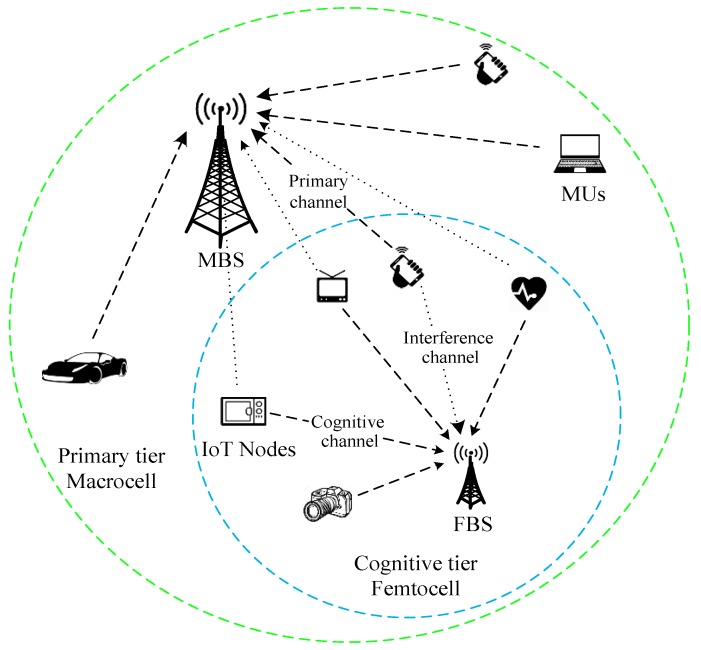
The two-tier cognitive network with coexisting macrocell and femtocell.

As shown in Figure 1, in compliance with the Fourth-Generation (4G) standard, such as LTE-A [21], we consider the uplink of a cognitive co-existing macrocell and IoT femtocell network, and assume that the transmissions are operated in the time-division duplex (TDD) mode. In the macrocell (primary tier), there is an $N_{R,p}^{}$ antenna MBS (as primary receiver) serving several MUs through single-carrier frequency division multiple access (SC-FDMA) [22]. The femtocell (cognitive tier) is comprised of an FBS (as cognitive receiver) and several IoT nodes distributed over the coverage area. For matters of spectral efficiency, the FBS is equipped with multiple antennas and an IA is introduced to enable space division multiplexing. As in tiered cognitive radio networks, the primary tier (macrocell) is oblivious to the cognitive tier (femtocell), and does not change its optimal precoding design; the cognitive tier should adapt its transmission to protect the primary tier from any undesired performance degradation. Hence, these two tiers are completely independent without any cooperation being established, and no cross-tier interference management strategy needs to be implemented at the primary tier. Since the macrocell operates in SC-FDMA, when accessing a certain licensed band, the femtocell is supposed to consider just one macrocell user (MU) in this band. However, this assumption does not decrease the generality of the proposed schemes. An extension to the model with multi-MUs per channel could be obtained by means of multiuser scheduling techniques [23] once the solution for the single MU case has been identified. 

More specifically, a *K*-user cognitive femtocell shares the licensed band with an MU simultaneously. There are $N_{T,k}^{}$ and $N_{R,k}^{}$ antennas at the *k*-th transmitter and the receiver in the femtocell, respectively, and a single antenna is equipped at the MU. We first focus on the full-IA algorithm without considering the cross-tier interference. When the MU is absent, the signal of the *k*-th IoT node can be recovered by full-IA at the FBS as:

(1) $$y_{k}=U_{k}^{H}H_{k,k}V_{k}x_{k}+\sum_{i=1,i\neq k}^{K}U_{k}^{H}H_{k,i}V_{i}x_{i}+U_{k}^{H}n_{k}.$$

Here, $x_{k}\in {\mathbb{C}}^{d_{k}\times 1}$ is the data of the IoT node, *k*, with the transmit power constraint, $P_{k}$, i.e., $E\left[{\left\|x_{k}\right\|}^{2}\right]=P_{k}$. $V_{k}\in {\mathbb{C}}^{N_{T,k}\times d_{k}}$ and $U_{k}\in {\mathbb{C}}^{N_{R,k}\times d_{k}}$ are the full-IA precoding and decoding matrices, respectively. $d_{k}$ is the number of data streams, and $n_{k}\in {\mathbb{C}}^{N_{R,k}\times 1}\sim CN(0,\sigma_{n}^{2}I_{N_{R,k}})$ is the additive white Gaussian noise vectors with a variance, $\sigma_{n}^{2}$. For the Rayleigh channel, let $H_{k,k}\in {\mathbb{C}}^{N_{R,k}\times N_{T,k}}$ denote the *k*-th transmission channel matrix, and $H_{k,i}\in {\mathbb{C}}^{N_{R,k}\times N_{T,i}}$ denote the interference channel matrix from the *i*-th transmitter to the *k*-th receiver. The entries of the Rayleigh channel matrix are standard independent identically distributed (i.i.d.) with zero-mean and unit-variance.

According to the full-IA algorithm, the co-tier interference among IoT nodes can be eliminated perfectly. Hence, the precoding and decoding matrices for each IoT node should be designed to satisfy the following conditions simultaneously [24]:

(2) $$U_{k}^{H}H_{k,i}V_{i}=0_{d_{k}\times d_{i}}\;k\neq i$$

(3) $$\mathrm{rank}\left\{U_{k}^{H}H_{k,k}V_{k}\right\}=d_{k},\;for\;k=1,2,\cdots K.$$

Condition (2) guarantees that the interference signals of unintended transmitters can be projected into the orthogonal subspace of the *k*-th receiver by $U_{k}^{}$ and $V_{i}$. Condition (3) preserves the required interference-free degree of freedom (DoF) for the desired signal of each IoT node. In the Rayleigh fading channel where the entries are independently distributed with no special structure, Condition (3) would be satisfied naturally when Condition (2) is satisfied [25]. If the feasibility condition is held for the tiered network, the received signal of the IoT node *k* can be given as:

(4) $$y_{k}=U_{k}^{H}H_{k,k}V_{k}x_{k}+U_{k}^{H}n_{k}.$$

Therefore, the transmission data rate of the *k*-th IoT node can be expressed as:

(5) $$R_{k}=\log_{2}\left|I_{d_{k}}+\frac{P_{k}}{d_{k}\sigma_{n}^{2}}\cdot \frac{U_{k}^{H}H_{k,k}V_{k}V_{k}^{K}H_{k,k}^{H}U_{k}}{U_{k}^{H}\left(I_{N_{R,k}}+Q_{k}\right)U_{k}}\right|,$$

where $Q_{k}$ is the co-tier interference covariance. Note that if IA is fully achieved, the interference covariance, $Q_{k}$, would be a zero matrix and the sum rate of IoT nodes can achieve its maximum by transmit power water-filling [26]:

(6) $$Q_{k}=\sum_{i=1,i\neq k}^{K}\frac{P_{i}}{d_{i}\sigma_{n}^{2}}H_{k,i}V_{i}V_{i}^{H}H_{k,i}^{H}\;$$

However, when the macrocell is active, since the MU will not sacrifice its own transmission rate to satisfy the IA conditions of IoT nodes, the sum rate of the entire network would be severely decreased. The received signals of both the macrocell and the femtocell would be disrupted by the cross-tier interference as:

(7) $$y_{p}=\overset{\mathrm{desired}\;\mathrm{signal}}{\overset{︷}{\overset{}{U_{p}^{H}h_{p}x_{p}}}}+\overset{\mathrm{cognitive}\;\mathrm{interference}}{\overset{︷}{\sum_{k=1}^{K}U_{p}^{H}G_{p,k}V_{k}x_{k}}}+\overset{\mathrm{noise}}{\overset{︷}{\overset{}{U_{p}^{H}n_{p}}}},$$

(8) $$y_{k}=\overset{\mathrm{desired}\;\mathrm{signal}}{\overset{︷}{\overset{}{U_{k}^{H}H_{k,k}V_{k}x_{k}}}}+\overset{\mathrm{co-tier}\;\mathrm{interference}}{\overset{︷}{\sum_{i=1,i\neq k}^{K}U_{k}^{H}H_{k,i}V_{i}x_{i}}}+\overset{\;\mathrm{cross-tier}\;\mathrm{interference}}{\overset{︷}{U_{k}^{H}g_{k,p}x_{p}}}+\overset{\mathrm{noise}}{\overset{︷}{U_{k}^{H}n_{k}}},$$

where $h_{p}$ is the channel coefficient vector between the MU and MBS. $G_{p,k}$ and $g_{k,p}$ are the interference matrix/vector between the two tiers. If the transmission power constraint of the MU is $P_{p}$, even with the co-tier interference being eliminated by full-IA, the data rate of the *k*-th IoT node is degraded as:

(9) $${R^{\prime }}_{k}=\log_{2}\left|I_{d_{k}}+\frac{P_{k}}{d_{k}}\cdot \frac{U_{k}^{H}H_{k,k}V_{k}V_{k}^{K}H_{k,k}^{H}U_{k}}{U_{k}^{H}\left(\sigma_{n}^{2}I_{N_{R,k}}+P_{p}g_{k,p}g_{k,p}^{H}\right)U_{k}}\right|.$$

The average received SINR of each data stream is also decreased by $10\mathrm{lg}\left(1+P_{p}/\sigma_{n}^{2}\right)$ dB [27]. As can be seen, the performance of the full-IA algorithm will be severely degraded by the cross-tier interference induced by cognitive spectrum sharing. Hence, the precoding schemes should be redesigned as cognitive empowered IA to mitigate the cross-tier interference and achieve a better network sum rate.

## 3. Cognitive Interference Alignment Schemes Design

In this section, two self-organizing cognitive IA schemes are proposed to mitigate the cross-tier interference caused by the cognitive spectrum sharing in an autonomous way. First, we consider the strict interference-free cognitive scenario and propose an interference nulling based cognitive IA scheme. In such a scheme, both co-tier and cross-tier interferences can be perfectly eliminated by aligning them into the orthogonal subspace of the intended signal. On the other hand, the interference nulling based IA algorithm can achieve its optimum only with negligible noise. Hence, when the interference-free constraint imposed on the femtocell users can be relaxed, we also propose a partial cognitive IA scheme that further improves the network performance under low and intermediate SNR conditions.

### 3.1. Cognitive Interference Elimination

According to the cognitive radio based paradigm [5], the cognitive network should first protect the primary user from the cognitive interference caused by spectrum sharing, rather than selfishly design its precoding scheme. In this subsection, we consider the proposal of a linear precoder design for the femtocell to nullify the cognitive interference of (7) in a totally autonomous way.

By looking at (7), the cognitive interference constraint should be satisfied as:

(10) $$\sum_{k=1}^{K}U_{p}^{H}G_{p,k}V_{k}x_{k}=0.\;$$

Note that one possible way to obtain the channel state information (CSI), for instance the cognitive interference channel, $G_{p,k}$, has been proposed in [28]. In such a method, the duality of TDD signals and the sounding reference in the LTE/LTE-A frame is exploited to estimate the channel. 

The data transmitted by the macrocell is unknown to the femtocell, which disqualifies the interference cancellation algorithms, such as interweave IA [29] or dirty paper coding. Furthermore, since the macrocell does not change its optimal decoding design, techniques that rely on cooperative coding between the network tiers are not implementable [17]. Self-configuring and self-optimizing precoding procedures should be designed for the femtocell. Assuming that, for the *k*-th IoT node, the precoder is divided to $V_{k}=W_{k}{\tilde{V}}_{k}$, then (10) can be rewritten as:

(11) $$G_{p}W\tilde{V}=0,\;$$

where $G_{p}$ is the aggregated interference channel as:

(12) $$G_{p}=\left[G_{p,1},G_{p,2},\cdots ,G_{p,K}\right]\in {\mathbb{C}}^{N_{R,p}\times \sum_{k=1}^{K}N_{T,k}}.$$

The aggregated precoder, W, in (11) is the direct sum [30] of the total K precoders:

(13) $$W=\overset{K}{\underset{k=1}{⊕}}W_{k},$$

where $W_{k}$ is the linear precoder that aims to fully eliminate the cognitive interference to the macrocell. 

It is straight-forward to see when the interference constraint (10) is always satisfied. Note that the macrocell does not need to cooperate or share any transmit information to create the aggregated precoder, W, which results in a lower backhaul transmission:

(14) $$G_{p,k}W_{k}=0,\;for\;k=1,2,\cdots ,K,$$

Now, we focus on the *k*-th IoT node to devise $W_{k}$. By looking into (14), we can see that the precoder must lie within the kernel of $G_{p,k}$. Here, we exploit the LQ decomposition to achieve a kernel space with lower computational complexity. Let $G_{p,k}=L_{k}Q_{k}$ be the LQ decomposition of the interference channel matrix, where $L_{k}\in {\mathbb{C}}^{N_{R,p}\times N_{T,k}}$ is a lower triangular matrix and $Q_{k}\in {\mathbb{C}}^{N_{T,k}\times N_{T,k}}$ is a unitary matrix. The matrix, $Q_{k}$, can be given in columns as $\left[q_{1},q_{2},\cdots ,q_{N_{T,k}}\right],$ and the last $N=N_{T,k}-N_{R,p}$ orthonormal vectors of $Q_{k}$ span the kernel space, $\ker\left(G_{p,k}\right)$. Therefore, the linear precoder, $W_{k}$, at the *k*-th IoT node can be defined as:

(15) $$W_{k}≜\left[q_{N+1},q_{N+2},\cdots ,q_{N_{T,k}}\right],$$

This fulfills the cognitive interference constraint (10). Note that, if the IoT nodes can obtain the channel vector, $h_{p}$, of the macrocell with the help of X2 interface [31], $W_{k}$ can be designed directly as the orthonormal basis of $h_{p}$, which may preserve more interference-free DoF for the network.

After eliminating the cognitive interference, we focus on the received signal of the MBS in the macrocell. By entering (15) into (7), we can obtain:

(16) $$y_{p}=U_{p}^{H}h_{p}x_{p}+{\tilde{n}}_{p}.$$

where ${\tilde{n}}_{p}=U_{p}^{H}n_{p}$ is the equivalent thermal noise, having the same size and statistic with the white Gaussian noise. It is shown that with the linear precoder, $W_{k}$, at IoT nodes, the macrocell is now free of cognitive interference, and the optimal decoding matrix for (16) can be expressed as:

(17) $$U_{p}^{}={\left(h_{p}^{†}\right)}^{H}=h_{p}^{}{\left(h_{p}^{H}h_{p}^{}\right)}^{-1},$$

Which is also known as the Moore-Penrose inverse of the channel vector.

### 3.2. Interference Nulling Based Cognitive IA

With the cognitive interference in (7) being fully eliminated by the linear precoder, we next consider the communication in the femtocell and attempt to mitigate both the co-tier and cross-tier interference for the precoding design. 

After designing the precoder as $V_{k}=W_{k}{\tilde{V}}_{k}$, the received signal in (8) can be simplified by introducing ${\tilde{H}}_{k,i}=H_{k,i}W_{i}$ as:

(18) $$y_{k}=U_{k}^{H}{\tilde{H}}_{k,k}{\tilde{V}}_{k}x_{k}+\sum_{i=1,i\neq k}^{K}U_{k}^{H}{\tilde{H}}_{k,i}{\tilde{V}}_{i}x_{i}+U_{k}^{H}g_{k,p}x_{p}+{\tilde{n}}_{k}.$$

The linear precoder, $W_{k}$, is constructed from the unitary matrix, $Q_{k}$, hence, the entries of ${\tilde{H}}_{k,i}$ remain the same statistic and can still be modeled as the Rayleigh fading channel. As we can only design the precoding and decoding matrices of the femtocell rather than changing the optimal transmission strategies of the macrocell, we consider mitigating the co-tier and cross-tier interferences with IA in an iterative way. By exploiting the channel propagation duality [32], the precoding and decoding matrices can be obtained via iterations between the interference channel and its reciprocal channel. To simplify the presentation, we summarize the interference nulling based cognitive IA in Algorithm 1.

More specifically, in the original channel, the optimization problem to minimize the interference power imposed on the *k*-th IoT node can be given as:

(19) $$\begin{array}{l} U_{k}^{*}=\arg\underset{U_{k}}{\min}P_{I,k}=\arg\min\mathrm{tr}\left\{U_{k}^{H}O_{k}U_{k}\right\}\\ s.t.\;U_{k}^{H}U_{k}=I_{N_{R,k}},\;for\;k=1,2,\cdots ,K, \end{array}$$

where the interference covariance matrix:

(20) $$O_{k}=\sum_{i=1,i\neq k}^{K}P_{i}{\tilde{H}}_{k,i}{\tilde{V}}_{i}{\tilde{V}}_{i}^{H}{\tilde{H}}_{k,i}^{H}+P_{p}g_{k,p}g_{k,p}^{H}.$$

The interference channel, $g_{k,p}$, in (20) should be estimated at the FBS by the blind channel estimation [33], which can obtain the CSI with insufficient or even no pilot. On the other hand, for the reciprocal channel, the optimization problem over the signal of the *k*-th IoT node can be expressed as:

(21) $$\begin{array}{l} U_{k}^{*}=\arg\underset{{\overset{\leftarrow }{U}}_{k}}{\min}\left[{\overset{\leftarrow }{P}}_{I,k}=\mathrm{tr}\left\{{\overset{\leftarrow }{U}}_{k}^{H}{\overset{\leftarrow }{O}}_{k}{\overset{\leftarrow }{U}}_{k}\right\}\right]\\ s.t.\;{\overset{\leftarrow }{U}}_{k}^{H}{\overset{\leftarrow }{U}}_{k}=I_{N_{R,k}},\;for\;k=1,2,\cdots ,K, \end{array}$$

where the reciprocal interference covariance matrix:

(22) $${\overset{\leftarrow }{O}}_{k}=\sum_{i=1,i\neq k}^{K}P_{i}{\overset{\leftarrow }{H}}_{i,k}{\overset{\leftarrow }{V}}_{i}{\overset{\leftarrow }{V}}_{i}^{H}{\overset{\leftarrow }{H}}_{i,k}^{H}.$$

Here, the corresponding variables in the reciprocal channel are denoted with a left arrow on top, i.e., ${\overset{\leftarrow }{U}}_{k}={\tilde{V}}_{k}$,${\overset{\leftarrow }{V}}_{k}=U_{k}$ and ${\overset{\leftarrow }{H}}_{i,k}={\tilde{H}}_{k,i}^{H}$. It should be noted that the cognitive interference on the macrocell is not involved in the reciprocal channel, since it has been eliminated by the linear precoder, $W_{k}$. 

The iterations alternate between the original and reciprocal channels, and in each iteration the receivers optimize their decoding matrix, ${\overset{}{U}}_{k}$ or ${\overset{\leftarrow }{U}}_{k}$. To null the overall interference in (19), the FBS should project the received signal into the subspace spanned by the eigenvectors corresponding to the smallest $d_{k}$ eigenvalues of $O_{k}$. Hence, in the original channel, the decoder, $U_{k}$, can be expressed as:

(23) $$U_{k}=\left[v_{O_{k},1},v_{O_{k},2},\cdots ,v_{O_{k},d_{k}}\right],$$

Whose columns are the first $d_{k}$ eigenvectors of $O_{k}$ in ascending order. In the reciprocal channel, we update the precoder, ${\overset{\leftarrow }{V}}_{k}$, with the $U_{k}$ determined in the original channel, then the reciprocal decoder, ${\overset{\leftarrow }{U}}_{k}$, can be obtained similarly as:

(24) $${\overset{\leftarrow }{U}}_{k}=\left[v_{{\overset{\leftarrow }{O}}_{k},1},v_{{\overset{\leftarrow }{O}}_{k},2},\cdots ,v_{{\overset{\leftarrow }{O}}_{k},d_{k}}\right],$$

where ${\overset{\leftarrow }{U}}_{k}$ is composed of the $d_{k}$ eigenvectors of the reciprocal interference covariance, ${\overset{\leftarrow }{O}}_{k}$. This process iterates in such a way until it converges.

**Algorithm 1** Interference nulling based cognitive interference alignment (IN-CIA) ($H_{k,i}$, $G_{p,k}$, $g_{k,p}$, $P_{k}$)
**1. Input:**
$H_{k,i}$: Co-tier channel; $g_{k,p}$: Cross-tier channel;$G_{p,k}$: Cognitive interference channel; $P_{k}$: Transmit power.
**2. Step I:**
Calculate LQ decomposition of $G_{p,k}=L_{k}Q_{k}$ for k=1, 2, ⋯, K.Construct the linear precoder $W_{k}$ as the last *N* columns of the unitary matrix $Q_{k}$ as (15).
**3. Initialize:**
Let ${\tilde{H}}_{k,i}=H_{k,i}W_{i}$, start with arbitrary unitary matrix ${\tilde{V}}_{k}$ that satisfies ${\tilde{V}}_{k}{\tilde{V}}_{k}^{H}=I_{d}$ for each IoT node.
**4. Step II:**

 **4.1 Original:**Compute interference covariance matrix in original channel as:$$O_{k}=\sum_{i=1,i\neq k}^{K}P_{k}{\tilde{H}}_{k,i}{\tilde{V}}_{i}{\tilde{V}}_{i}^{H}{\tilde{H}}_{k,i}^{H}+P_{p}g_{k,p}g_{k,p}^{H}.$$ **4.2 Design $U_{k}$:**Compute the original decoder at the *k*-th receiver as the first $d_{k}$ eigenvectors of $O_{k}$:$$U_{k}=\left[v_{O_{k},1},\;v_{O_{k},2},\;\cdots ,\;v_{O_{k},d_{k}}\right],\;for\;k=1,\;2,\;\cdots ,\;K.$$ **4.3 Reciprocal:**Consider the reciprocal channel and update all the precoder ${\overset{\leftarrow }{V}}_{k}=U_{k}$ for k=1,⋯,K. Then compute reciprocal interference covariance matrix as:$${\overset{\leftarrow }{O}}_{k}=\sum_{i=1,i\neq k}^{K}P_{k}{\overset{\leftarrow }{H}}_{i,k}{\overset{\leftarrow }{V}}_{i}{\overset{\leftarrow }{V}}_{i}^{H}{\overset{\leftarrow }{H}}_{i,k}^{H}.$$ **4.4 Design ${\overset{\leftarrow }{U}}_{k}$:**Similarly, compute the reciprocal decoder at the *k*-th reversed receiver as the first $d_{k}$ eigenvectors of $O_{k}$:$${\overset{\leftarrow }{U}}_{k}=\left[v_{{\overset{\leftarrow }{O}}_{k},1},v_{{\overset{\leftarrow }{O}}_{k},2},\cdots ,v_{{\overset{\leftarrow }{O}}_{k},d_{k}}\right],\;for\;k=1,\;2,\;\cdots ,\;K.$$
**5. Reverse**
Reverse the communication channel and update the decoder as ${\tilde{V}}_{k}={\overset{\leftarrow }{U}}_{k}$, for k=1, 2, ⋯, K.
**6. Loop:**
**If**$\max\left\{\left\|{\overset{\leftarrow }{V}}_{k}-U_{k}\right\|,\left\|{\overset{\leftarrow }{U}}_{k}-{\tilde{V}}_{k}\right\|\right\}\geq \varepsilon_{k}$, i.e., the momentum greater than a given threshold.**Jump to** Step 2.
**7. Converge:**
**Else**: **Return** the precoder and decoder $V_{k}=W_{k}{\tilde{V}}_{k}$ and $U_{k}$.

### 3.3. Partial Cognitive IA

The interference nulling based IA algorithm achieves its optimum only with negligible noise. In this sub section, we consider improving the performance interference nulling based IA algorithm under low and intermediate SNR conditions and propose a partial cognitive IA scheme.

The interference nulling based cognitive IA aims to perfectly align both co-tier and cross-tier interferences, providing a totally interference-free transmission for the femtocell users. However, the full-IA method considers just the interference nulling rather than capacity optimization, and achieves its optimum only in the networks with rather high SNR. When the transmit power is limited, the orthogonal precoder makes no attempt to suppress the noise or maximize the desired signal, which is generally a suboptimal solution with low or intermediate SNR values. The performance of the cognitive IA can be further enhanced by partially aligning the interference. Hence, we propose the partial cognitive IA to jointly consider the signal, interference, and noise power, and maximize the transmission rate directly. The steps of the partial cognitive IA scheme are summarized in Algorithm 2.

**Algorithm 2** Partial cognitive IA (P-CIA) ($H_{k,k}$, $H_{k,i}$, $G_{p,k}$, $g_{k,p}$, $P_{k}$, $\sigma_{n}$)
**1. Input:**
$H_{k,k}$: Transmission channel; $g_{k,p}$: Cross-tier channel;$H_{k,i}$: Co-tier channel; $\sigma_{n}$: White noise power;$G_{p,k}$: Cognitive interference channel; $P_{k}$: Transmit power.
**2. Step I:**
Calculate LQ decomposition of $G_{p,k}=L_{k}Q_{k}$, k=1, ⋯, K.Construct the linear precoder $W_{k}$ as the last *N* columns of the unitary matrix $Q_{k}$ as (15).
**3. Initialize:**
Let ${\tilde{H}}_{k,i}=H_{k,i}W_{i}$, initialize with arbitrary unitary matrix ${\tilde{V}}_{k}=\left\{{\tilde{v}}_{k,1},\cdots ,{\tilde{v}}_{k,d_{s}}\right\},$
$U_{k}=\left\{u_{k,1},\cdots ,u_{k,d_{s}}\right\},$ whose columns are linearly independent unit vectors.
**4. Step II:**
Formulate the optimization problem for the *s*-th data stream of the *k*-th node as:$$\begin{array}{l} u_{k,s}^{*}=\arg\max\;\log_{2}\left|1+\frac{P_{k}}{d_{k}}\cdot \frac{u_{k,s}{\tilde{H}}_{k,k}{\tilde{v}}_{k,s}{\tilde{v}}_{k,s}^{H}{\tilde{H}}_{k,k}^{H}u_{k,s}^{H}}{u_{k,s}O_{k,s}u_{k,s}^{H}}\right|,\\ s.t.\;u_{k,s}^{H}u_{k,s}=1,\;for\;\forall k\in \left[1,K\right],\;s\in \left[1,d_{k}\right], \end{array}$$ **4.1 Original:**where the interference plus noise covariance matrix $O_{k,s}$ is:$$O_{k,s}=\sum_{i=1}^{K}\frac{P_{i}}{d_{i}}\sum_{\begin{array}{c} j=1\\ j\neq s,for\;i=k \end{array}}^{d_{i}}{\tilde{H}}_{k,i}{\tilde{v}}_{i,j}{\tilde{v}}_{i,j}^{H}{\tilde{H}}_{k,i}^{H}+P_{p}g_{k,p}g_{k,p}^{H}+\sigma_{n}^{2}I_{N_{R,k}}.$$ **4.2 Design $u_{k,s}^{}$:**Calculate the optimal receiving vector for the problem as:$$u_{k,s}^{*}=\frac{O_{k,s}^{-1}{\tilde{H}}_{k,k}{\tilde{v}}_{k,s}}{\left\|O_{k,s}^{-1}{\tilde{H}}_{k,k}{\tilde{v}}_{k,s}\right\|}.$$ **4.3 Reciprocal:**In reciprocal channel, update all the precoder ${\overset{\leftarrow }{v}}_{k,s}=u_{k,s}^{*}$ for ∀k∈[1,K] and $\forall s\in \left[1,d_{k}\right].$ Compute reciprocal interference plus noise covariance matrix as:$${\overset{\leftarrow }{O}}_{k,s}=\sum_{i=1}^{K}\frac{P_{i}}{d_{i}}\sum_{\begin{array}{c} j=1\\ j\neq s,for\;i=k \end{array}}^{d_{i}}{\overset{\leftarrow }{H}}_{k,i}{\overset{\leftarrow }{v}}_{i,j}{\overset{\leftarrow }{v}}_{i,j}^{H}{\overset{\leftarrow }{H}}_{k,i}^{H}+\sigma_{n}^{2}I_{N_{T,k}}.$$ **4.4 Design ${\overset{\leftarrow }{u}}_{k,s}^{}$:**Similarly, the optimal receiving vector for reciprocal channel is given by:$${\overset{\leftarrow }{u}}_{k,s}^{*}=\frac{{\overset{\leftarrow }{O}}_{k,s}^{-1}{\overset{\leftarrow }{H}}_{k,k}{\overset{\leftarrow }{v}}_{k,s}}{\left\|{\overset{\leftarrow }{O}}_{k,s}^{-1}{\overset{\leftarrow }{H}}_{k,k}{\overset{\leftarrow }{v}}_{k,s}\right\|}.$$
Reverse the communication channel and update all the decoder as ${\tilde{v}}_{k,s}={\overset{\leftarrow }{u}}_{k,s}^{*}$, for $\forall k\in \left[1,K\right],\;\forall s\in \left[1,d_{k}\right].$
**5. Loop:**
**If**$\max\left\{\left\|{\overset{\leftarrow }{V}}_{k}-U_{k}\right\|,\left\|{\overset{\leftarrow }{U}}_{k}-{\tilde{V}}_{k}\right\|\right\}\geq \varepsilon_{k}$, i.e., the momentum greater than a given threshold**Jump to** Step 2.
**6. Converge:**
**Else**: **Return** the coding matrices $V_{k}=W_{k}{\tilde{V}}_{k}$ and $U_{k}$.

The proposed partial cognitive IA also exploits the channel propagation duality and operates in an iterative manner. We identify $U_{k}$ and ${\overset{\leftarrow }{U}}_{k}$ to be the decoders at the *k*-th receiver in the original and reciprocal channels, respectively. Instead of eliminating the interference completely, we consider both the noise and interference power for each IoT node, aiming to maximize the transmission rate of the data streams directly. More specifically, the optimization problem for the *s*-th data stream of the IoT node, *k*, in the original channel can be expressed as:

(25) $$\begin{array}{l} u_{k,s}^{*}=\arg\max\;\log_{2}\left|1+\frac{P_{k}}{d_{k}}\cdot \frac{u_{k,s}{\tilde{H}}_{k,k}{\tilde{v}}_{k,s}{\tilde{v}}_{k,s}^{H}{\tilde{H}}_{k,k}^{H}u_{k,s}^{H}}{u_{k,s}O_{k,s}u_{k,s}^{H}}\right|,\\ s.t.\;u_{k,s}^{H}u_{k,s}=1,\;for\;\forall k\in \left[1,K\right],\;\forall s\in \left[1,d_{k}\right], \end{array}$$

where the vectors, $u_{k,s}$ and ${\tilde{v}}_{k,s}$, are the *s*-th column of the coding matrices, $U_{k}$ and ${\tilde{V}}_{k}$, respectively. In (25), the interference and noise covariance matrix, $O_{k,s}$, is represented as:

(26) $$O_{k,s}=\sum_{i=1}^{K}\frac{P_{i}}{d_{i}}\sum_{\begin{array}{c} j=1\\ j\neq s,for\;i=k \end{array}}^{d_{i}}{\tilde{H}}_{k,i}{\tilde{v}}_{i,j}{\tilde{v}}_{i,j}^{H}{\tilde{H}}_{k,i}^{H}+P_{p}g_{k,p}g_{k,p}^{H}+\sigma_{n}^{2}I_{N_{R,k}}.$$

It is known that, for the given interference and noise covariance, $O_{k,l}$, and the precoding vector, ${\tilde{v}}_{k,s}$, the optimal decoding vector to problem (25) is given by [34]:

(27) $$u_{k,s}^{*}=\frac{O_{k,s}^{-1}{\tilde{H}}_{k,k}{\tilde{v}}_{k,s}}{\left\|O_{k,s}^{-1}{\tilde{H}}_{k,k}{\tilde{v}}_{k,s}\right\|}.$$

For the reciprocal channel, the partial cognitive IA algorithm operates in a similar way. We update the reciprocal precoder, ${\overset{\leftarrow }{V}}_{k}$, with $U_{k}$ as ${\overset{\leftarrow }{V}}_{k}=U_{k}$, and the reciprocal interference and noise covariance is constructed without involving the cognitive interference to the macrocell:

(28) $${\overset{\leftarrow }{O}}_{k,s}=\sum_{i=1}^{K}\frac{P_{i}}{d_{i}}\sum_{\begin{array}{c} j=1\\ j\neq s,for\;i=k \end{array}}^{d_{i}}{\overset{\leftarrow }{H}}_{k,i}{\overset{\leftarrow }{v}}_{i,j}{\overset{\leftarrow }{v}}_{i,j}^{H}{\overset{\leftarrow }{H}}_{k,i}^{H}+\sigma_{n}^{2}I_{N_{T,k}}.$$

The optimization problem of the receiving vector, ${\overset{\leftarrow }{u}}_{k,s}$, in the reciprocal channel can be solved in the same way as:

(29) $${\overset{\leftarrow }{u}}_{k,s}^{*}=\frac{{\overset{\leftarrow }{O}}_{k,s}^{-1}{\overset{\leftarrow }{H}}_{k,k}{\overset{\leftarrow }{v}}_{k,s}}{\left\|{\overset{\leftarrow }{O}}_{k,s}^{-1}{\overset{\leftarrow }{H}}_{k,k}{\overset{\leftarrow }{v}}_{k,s}\right\|}.$$

The procedure of the partial cognitive IA algorithm also iterates between (27) and (29) until the values of $U_{k}$ and ${\overset{\leftarrow }{U}}_{k}$ converge.

## 4. Performance Analyses of the Cognitive IA Schemes

In this section, we provide the proof of convergence, capacity, and feasibility analyses to show the effectiveness of the proposed cognitive IA schemes.

### 4.1. Proof of Convergence

In this subsection, we illustrate how the proposed iterative algorithms are proved to be convergent. Without a loss of generality, we assume the transmit power of each transmitter is normalized. In each iteration of the interference nulling based cognitive IA scheme, the decoder, $U_{k}/{\overset{\leftarrow }{U}}_{k}$, is designed to nullify the original/reciprocal interference power imposed on each IoT node, which is associated with the covariance matrices in (21) and (22). More specifically, in the original channel, the decoder, $U_{k}$, is designed as (23) to minimize the overall interference power, $P_{I,k}.$ Since the decoders, $U_{k},\forall k\in \left[1,K\right]$, are uncoupled from each other and the sum interference power is given by $P_{I}=\sum_{k=1}^{K}P_{I,k}$, the objective function can be decoupled as:

(30) $$\underset{U_{1},U_{2},\cdots ,U_{K}}{\min}P_{I}=\underset{U_{1},U_{2},\cdots ,U_{K}}{\min}\sum_{k=1}^{K}P_{I,k}=\sum_{k=1}^{K}\left\{\underset{U_{k}}{\min}P_{I,k}\right\},$$

where:

(31) $$P_{I,k}=\sum_{i=1,i\neq k}^{K}\mathrm{tr}\left\{U_{k}^{H}{\tilde{H}}_{k,i}{\tilde{V}}_{i}{\tilde{V}}_{i}^{H}{\tilde{H}}_{k,i}^{H}U_{k}\right\}+\mathrm{tr}\left\{U_{k}^{H}g_{k,p}g_{k,p}^{H}U_{k}\right\}.$$

We know that, in each iteration of the original channel, it always chooses the optimal $\left\{U_{1},\;\cdots ,\;U_{K}\right\}$ to minimize the sum interference power in (30) and reduce the value of $P_{I}$.

In the reciprocal channel, the reversed decoder, ${\overset{\leftarrow }{U}}_{k}$, associated with the *k*-th IoT node is designed without considering the cross-tier interference from the macrocell. Hence, the reciprocal objective function of the *k*-th IoT node is given as:

(32) $${\overset{\leftarrow }{P}}_{I,k}=\sum_{i=1,i\neq k}^{K}\mathrm{tr}\left\{{\overset{\leftarrow }{U}}_{k}^{H}{\overset{\leftarrow }{H}}_{i,k}{\overset{\leftarrow }{V}}_{i}{\overset{\leftarrow }{V}}_{i}^{H}{\overset{\leftarrow }{H}}_{i,k}^{H}{\overset{\leftarrow }{U}}_{k}\right\}.\;$$

By substituting ${\overset{\leftarrow }{U}}_{k}={\tilde{V}}_{k}$, ${\overset{\leftarrow }{V}}_{k}=U_{k}$ and ${\overset{\leftarrow }{H}}_{i,k}={\tilde{H}}_{k,i}^{H}$ to (32), we get:

(33) $${\overset{\leftarrow }{P}}_{I,k}=\sum_{i=1,i\neq k}^{K}\mathrm{tr}\left\{{\overset{\leftarrow }{U}}_{k}^{H}{\overset{\leftarrow }{H}}_{i,k}{\overset{\leftarrow }{V}}_{i}{\overset{\leftarrow }{V}}_{i}^{H}{\overset{\leftarrow }{H}}_{i,k}^{H}{\overset{\leftarrow }{U}}_{k}\right\}.\;$$

As can be seen from (31) and (33), the first term of $P_{I,k}$ is equal to the reciprocal objective function, ${\overset{\leftarrow }{P}}_{I,k}$, while the second term of $P_{I,k}$ is not affected by the reversed precoder, ${\overset{\leftarrow }{U}}_{k}={\tilde{V}}_{k}$. It means that the optimal decoder that ensures a receiver suffers the least co-tier interference also causes the least interference to others in the reciprocal channel. Therefore, when the value of ${\overset{\leftarrow }{U}}_{k}$ is chosen to minimize ${\overset{\leftarrow }{P}}_{I,k}$, it also reduces the value of $P_{I,k}$. Since the value of the interference power decreases monotonically in every iteration and is lower bounded by zero, hence, convergence of the algorithm can be guaranteed. The convergence of the partial cognitive IA scheme can also be discussed in a similar way, and a semi-definite programming (SDP) problem generally requires $O[\sqrt{Kd}\log(1/\varepsilon )]$ iterations to converge with ε as the predefined solution accuracy.

### 4.2. Capacity Analysis

We next demonstrate the superiority of the proposed cognitive IA schemes by proving that it can preserve the same performance as the non-cognitive IA based network when they are both feasible to transmit the same number of data streams. 

With the proposed interference nulling based cognitive IA scheme, the cross-tier interference form the macrocell has been eliminated and we have:

(34) $$P_{p}U_{k}^{H}g_{k,p}g_{k,p}^{H}U_{k}=0.$$

Therefore, the data rate of the *k*-th IoT node coexisting with the macrocell can be expressed as:

(35) $$R_{k}^{CIA}=\log_{2}\left|I_{d_{k}}+\frac{P_{k}}{d_{k}\sigma_{n}^{2}}\cdot \frac{U_{k}^{H}{\tilde{H}}_{k,k}{\tilde{V}}_{k}{\tilde{V}}_{k}^{K}{\tilde{H}}_{k,k}^{H}U_{k}}{U_{k}^{H}U_{k}}\right|.\;$$

Recall that in full-IA based networks, the data rate of the *k*-th IoT node can be expressed as:

(36) $$R_{k}^{full-IA}=\log_{2}\left|I_{d_{k}}+\frac{P_{k}}{d_{k}\sigma_{n}^{2}}\cdot \frac{U_{k}^{H}H_{k,k}V_{k}V_{k}^{K}H_{k,k}^{H}U_{k}}{U_{k}^{H}\left(I_{N_{R,k}}+Q_{k}\right)U_{k}}\right|\;$$

By comparing (35) with (36) we can see when the interference covariance, $Q_{k}$, becomes a zero matrix when full-IA is achieved, and these two equations have the same form with just different dimensions of the channel matrices, $H_{k,k}$ and ${\tilde{H}}_{k,k}$. Note that all the precoders and decoders in both IA schemes are i.i.d. unitary matrices and are independent of the channel matrix, which means the coding process does not change the distribution of the channel matrix/vector. Therefore, the expectation of the channel gain remains the same, and the proposed cognitive IA scheme achieves the same average sum rate as the non-cognitive homogeneous IA with the same number of data streams, $d_{k}$, k=1, 2, ⋯, K. As the interference nulling based cognitive IA scheme becomes less effective under low and intermediate SNR, the proposed partial cognitive IA scheme aims to directly maximize the transmission rate for each data stream. Although the co-tier and cross-tier interferences are not perfectly aligned into the orthogonal subspace of the desired signal, the partial cognitive IA still achieves a better network sum rate than the interference nulling based cognitive IA scheme.

### 4.3. Feasibility Condition Analysis

The feasibility condition is also a primary concern in IA based schemes. In this subsection, we analyze the feasibility condition of the proposed cognitive IA schemes and show that more antennas are required to empower a network with cognition. 

Despite being organized in different forms, the two cognitive IA schemes hold the same feasibility condition. Inspired by the Bezout’s theorem, a generic polynomial system is solvable if, and only if, the number of variables is not below the number of equations. Thus, we count the number of cognitive IA equations and that of the unsolved variables to derive the feasibility condition. We first focus on the co-tier interference constraint in (2) with the equivalent channel matrices, ${\tilde{H}}_{k,i}$, k,i∈[1,K]. The total number of Equations in (2) is counted as:

(37) $$N_{e}^{IA}=\sum_{k,i\in \left[1,K\right],\;k\neq i}d_{k}\times d_{i}.\;$$

when counting the number of variables, we can see that not all the variables are mutually independent. After eliminating $2d_{k}^{2}$ superfluous variables for the *k*-th IoT node, the total number of variables can be given as:

(38) $$N_{v}^{IA}=\sum_{k=1}^{K}d_{k}\left(N_{T,k}+N_{R,k}-N_{R,p}-2d_{k}\right).\;$$

To simplify the calculations, we assume that the tiered network is a symmetrical one, i.e., $N_{T,k}=N_{T}$, $N_{R,k}=N_{R}$ and $d_{k}=d,$ for ∀k∈[1,K]. Note that the assumption of network symmetry does not limit the generality of the analysis. For asymmetrical networks, we can also derive the feasibility condition with Bezout’s theorem by comparing the total number of equations and unsolved variables in the whole network. 

The unsolved variables in both the precoder and decoder can be determined by the IA polynomials, and are solvable if, and only if, the number of the equations, $N_{e}^{IA}$, does not exceed that of the variables, $N_{v}^{IA}$, as:

(39) $$d^{2}K\left(K-1\right)\leq dK\left(N_{T}+N_{R}-N_{R,p}-2d\right),\;$$

Which means the maximum number of data streams has the limit:

(40) $$d\leq \frac{N_{T}+N_{R}-N_{R,p}}{K+1}.\;$$

When considering the cognitive interference, the following constraint should be satisfied as:

(41) $$U_{k}^{H}g_{k,p}=0\in {\mathbb{C}}^{d\times 1},\;for\;k=1,\;2,\;\cdots ,\;K.\;$$

Hence, the polynomial (41) is also involved to derive the feasibility condition of the proposed cognitive IA schemes. It can be seen that considering (41), there exists d independent equations for each IoT node, and the total number of the equations is $N_{e}^{CIA}=\sum_{k=1}^{K}d_{k}=Kd$. Similarly, we count the variables and remove the superfluous ones, hence, the total number of the variables is:

(42) $$N_{v}^{CIA}=\sum_{k=1}^{K}d_{k}\left(N_{R,k}-d_{k}\right)=Kd\left(N_{R}-d\right)\;$$

Since the $N_{v}^{CIA}$ variables can be solved only by the $N_{e}^{CIA}$ equations, and the decoder also needs to cooperate with the precoder to align the co-tier interference, it should satisfy the condition, $N_{v}^{CIA}\geq N_{e}^{CIA}+1$, i.e., $N_{R}\geq d+1$.

With the analysis above, we can obtain the total number of equations as $N_{e}=N_{e}^{IA}+N_{e}^{CIA}$, and the number of variables, $N_{v}^{IA}$, remains the same. The feasibility condition of the proposed cognitive IA schemes with d data streams for each IoT node can be represented as:

(43) $$\{\begin{array}{c} dK+d+1\leq N_{T}+N_{R}-N_{R,p},\\ d\leq \min\left\{N_{R}-1,\;N_{T}-N_{R,p}\right\}. \end{array}\;$$

Although the proposed cognitive IA schemes can achieve the same transmission rate as the full-IA homogeneous network, we should note that, in the femtocell, more antennas at the transmitters and receivers are required to deal with the interference introduced by the heterogeneous structure, as well as to make the cognitive IA schemes feasible. More specifically, we can empower a feasible IA-based network with cognition by adding $N_{R,p}-1$ antennas at each transmitter to eliminate the cognitive interference, and one more antenna at each receiver to align the cross-tier interference.

## 5. Numerical Simulation Results

In this section, the numerical results are presented. To evaluate the effectiveness of the proposed schemes, we compared the achieved sum rate of the IN-CIA and P-CIA algorithms with that of several existing precoding methods, as well as with different data streams and antenna configurations. In the simulations, we assumed there are three IoT nodes in the femtocell coexisting with a single antenna MU and a dual antenna MBS of the macrocell, and that identical transmit power is allocated for each user, i.e., K=3, $N_{T,p}=1$, $N_{R,p}=2$ and $P_{k}=P$, for k=p, 1, 2, 3. Each cognitive pair transmits d=1 data stream with $N_{T,k}=3$ transmitting antennas at the IoT node and $N_{R,k}=4$ receiving antennas at the MBS. 

**Figure 2:**
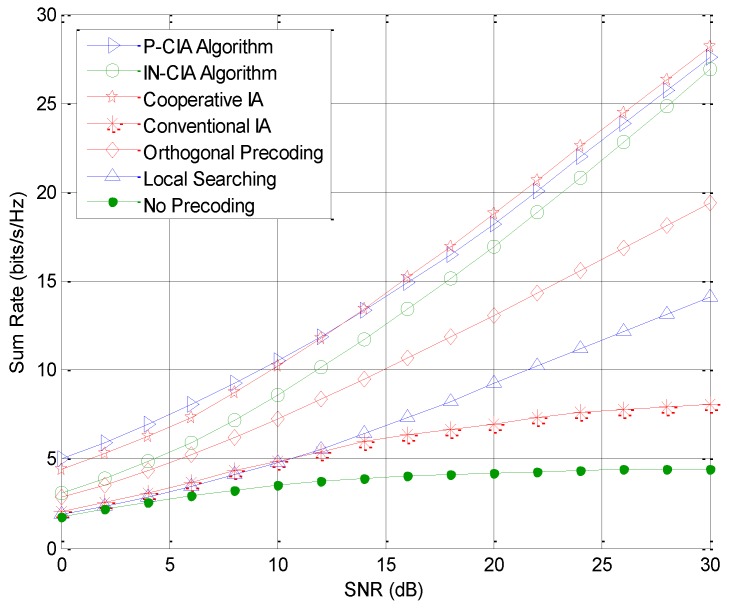
Sum rate comparison of the Internet of Things (IoT) oriented femtocell with existing precoding algorithms.

**Figure 3:**
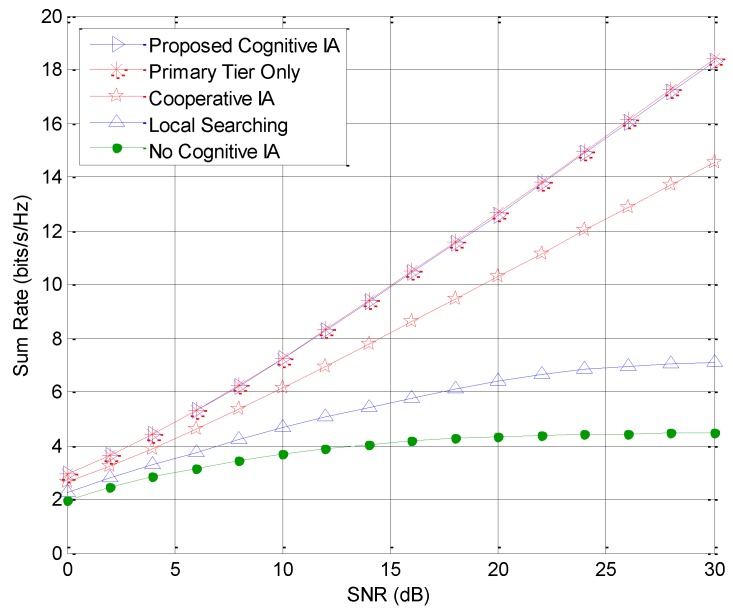
Sum rate comparison of the macrocell with different precoding algorithms.

Figure 2 shows the sum rate comparison of the IoT oriented femtocell with several existing interference suppression precoding algorithms. When the feasibility condition is satisfied, both the cooperative IA and P-CIA outperform the IN-CIA, and the performance converges in the high SNR region. This is because the cooperative IA achieves a slightly higher channel gain with the cooperation from the macrocell to eliminate the cross-tier interference, and the P-CIA further enhances the sum rate by jointly considering the desired channel gain, interference, and noise. It is also observed that the IA algorithms generally provide remarkable benefits over the orthogonality based precoding methods, since IA can obtain half of the degrees of freedom per channel use. In Figure 3, we compare the sum rate of the macrocell with several precoding algorithms. As the proposed cognitive IA schemes fully eliminate the cognitive interference caused to the macrocell, the MUs can achieve an almost identical performance with that of the non-cognitive homogeneous network. We can also see that the cooperative IA compromises the primary users in the macrocell to ensure better performance of the IoT oriented cognitive femtocell, which turns out to be a major limitation. The conventional non-cognitive IA algorithm that neglects the cognitive interference also causes significant performance degradation to MUs.

**Figure 4:**
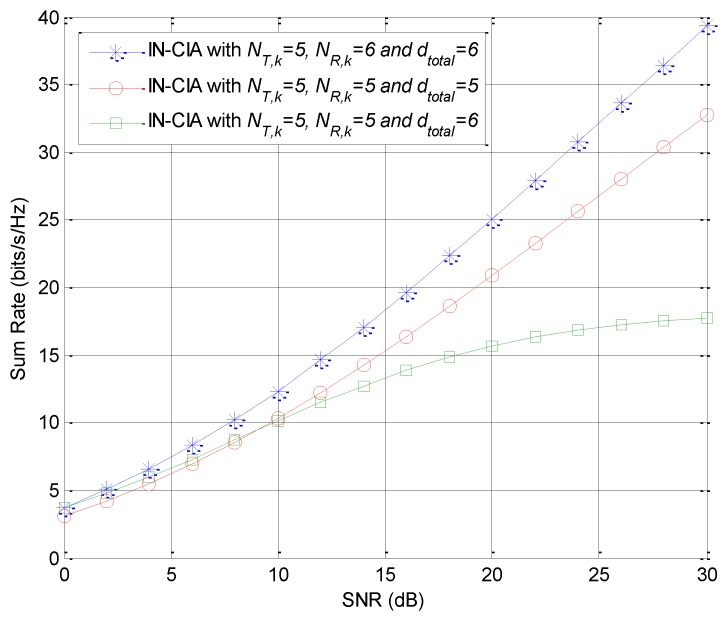
Sum-rate comparison with different numbers of data streams and antenna configurations.

Next, we consider scenarios with different numbers of data streams and antenna configurations to evaluate the performance of the proposed scheme. It can be seen from Figure 4 that the proposed cognitive IA algorithm can achieve the desired sum rate when the number of antenna meets the feasibility condition. However, the network performance would be severely degraded without enough antennas equipped as the feasibility condition requires. In such a case, to make the IA schemes feasible, we can either add more antennas at each transmitter/receiver or reduce the required degrees of freedom by cutting down the number of data streams in the network.

To provide measurement results from a practical perspective, we evaluated the percentage of interference leakage, which is defined as the fraction of the interference power in the dimensions reserved for the desired signal [17]. The interference leakage percentage at each user is given as:

(44) $$p_{k}=\frac{\sum_{i=1}^{d_{k}}\lambda_{O_{k},i}}{\mathrm{tr}\left\{O_{k}\right\}}\;\mathrm{for}\;k=1,\;\cdots ,\;K.$$

where $O_{k}$ denotes the interference covariance matrix in (20), and $\lambda_{O_{k},i}$ is the *i*-th eigenvalue of the matrix in ascending order. The numerator and the denominator of (44) are the interference and desired signal powers at the receiver, *k*, respectively.

**Figure 5:**
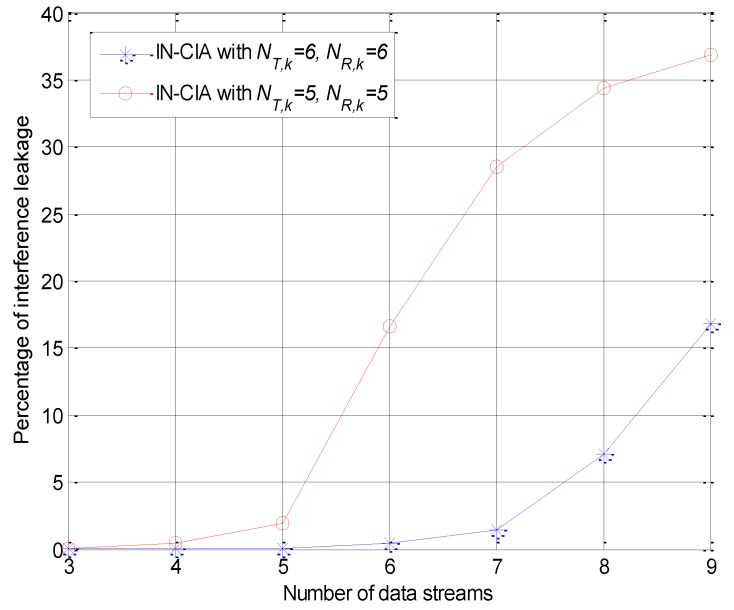
Percentage of interference leakage versus the number of data streams.

As can be seen from Figure 5, in the simulation, the expected total degrees of freedom for the two curves are 5 and 7, respectively. When the antenna configuration just satisfies the feasibility condition, the IA based network utilizes all the degrees of freedom to transmit data streams. Limited by the numerical errors of the iterative approach, the interference may not be perfectly aligned into the orthogonal subspace. However, the maximum percentage of interference leakage is less than 3% when it is feasible, which does not reduce the effectiveness of the proposed cognitive IA algorithm. If more strict interference-free transmissions are needed in the practical implementation, one or two degrees of freedom can be reserved to achieve more precise interference alignment.

## 6. Conclusions

In this paper, we targeted our efforts on IoT oriented heterogeneous networks with coexisting femtocell and macrocell. We aimed to manage the cognitive interference caused to macrocell as well as ensuring an optimal capacity of the femtocell, hence, two novel self-organized cognitive IA schemes were proposed. The interference nulling based cognitive IA scheme can perfectly eliminate both the co-tier and cross-tier interference of the femtocell by aligning them into the lower dimension, while preserving the interference-free transmission for the macrocell. As the interference nulling based IA algorithm can achieve its optimum only with negligible noise, we also presented a partial cognitive IA (P-CIA) scheme by loosening the co-tier interference constraint, and further improved the network performance under low and intermediate SNR conditions. Additionally, the feasibility condition, capacity, and convergence analyses were derived. Both the theoretical and numerical results demonstrated that the proposed cognitive IA schemes achieved a significant improvement on the network capacity while causing no performance degradation to the primary users, indicating that the cognitive IA has a better application prospect for IoT oriented heterogeneous networks.

In addition, in this paper, we mainly focused on the physical layer interference management with a stochastic geometry model. Extending the results of this paper to the network layer are interesting future avenues for our work. One possible approach is to exploit the multi-hop relay and explore corresponding routing protocols to further improve the network performance.
